# Preclinical studies of histotripsy for intracranial tumors

**DOI:** 10.3389/fneur.2025.1727225

**Published:** 2026-01-13

**Authors:** Hong Chen, Zhenbin Xu, Shengmin Zhang

**Affiliations:** Department of Ultrasound Medicine, The First Affiliated Hospital of Ningbo University, Ningbo, China

**Keywords:** ablation, blood–brain barrier opening, histotripsy, immunomodulation, intracranial tumors, non-invasive

## Abstract

The management of intracranial tumors, especially malignant or refractory types, remains a formidable clinical challenge due to the paucity of ideal therapeutic options. Histotripsy, an emerging ultrasound technology, presents a paradigm-shifting therapeutic avenue. As a noninvasive, nonthermal, and nonionizing ultrasonic tissue destruction technique, histotripsy has shown promising therapeutic effects in preclinical studies on intracranial tumors. Preclinical studies demonstrate the capability of histotripsy to achieve precise mechanical ablation of tumor tissue, concurrently minimizing hemorrhagic risk and collateral damage to surrounding healthy structures. Its efficacy can be monitored using MRI sequences or by leveraging the intrinsic acoustic cavitation emission signals. Beyond its direct ablative role, histotripsy can also enable the transient opening of the blood–brain barrier. Although histotripsy for the treatment of intracranial tumors is still in the preclinical research phase, with current studies focusing on validating its feasibility and safety, the available data have provided preliminary evidence of favorable therapeutic effects. Although challenges remain for its clinical translation, initial solutions have been proposed. Looking forward, with further research and technological optimization, histotripsy is expected to play a key role in the treatment of intracranial tumors and fully demonstrate its clinical application value.

## 1 Introduction

Intracranial tumors, particularly their malignant forms, are associated with a poor prognosis, presenting profound clinical management challenges that severely undermine patient quality of life and survival (1, 2). Conventional management—including invasive resection, radiotherapy, and systemic chemotherapy—is frequently constrained by collateral damage to neural tissues, radiation injury, and myelosuppression (3, 4). Furthermore, minimally invasive alternatives such as transarterial embolization, laser interstitial thermal therapy, and high-intensity focused ultrasound (HIFU) confront inherent limitations, including complex tumor vascular topography and risks of iatrogenic thermal injury (5–7). Within this challenging therapeutic landscape, histotripsy has emerged as a transformative non-thermal ablation modality that utilizes ultrasound mechanical effect for precise tissue disruption, offering a promising alternative to conventional paradigms.

Histotripsy is often confused with HIFU, but their mechanisms of action are fundamentally distinct (Table 1). HIFU uses continuous or long pulses to induce thermal coagulation of tissue (frequency 0.2–2 MHz, duty cycle high), requiring heat accumulation over a few hours (8). In contrast, Typical transcranial histotripsy usually employs short (1–2 cycles) ultrasound pulses (frequency 250 kHz-5 MHz, duty cycle <2%) to induce cavitation clouds that mechanically destroy cell structures, achieving precise tissue ablation within minutes through pulse repetition frequency control (9). This allows histotripsy to avoid the heat accumulation associated with thermal ablation, enabling “purely mechanical ablation.” Thermal ablation techniques, such as HIFU, are often compromised by the heat-sink effect. This phenomenon occurs when blood flow in major vessels adjacent to the target region dissipates thermal energy, preventing the tissue from reaching the critical temperature required for coagulative necrosis and thus undermining the ablation efficacy. In stark contrast, histotripsy—a purely mechanical non-thermal ablation modality—overcomes this fundamental limitation. Its mechanism of action, which relies on the mechanical fractionation of tissue via acoustic cavitation, is inherently independent of thermal energy accumulation or dissipation.

**Table 1:** The difference between HIFU and histotripsy [data is from the references (8, 9)].

Mechanism	HIFU	Histotripsy
Core mechanism	Thermal ablation	Mechanical cavitation
Mode of action	Conversion of acoustic energy into thermal energy, causing coagulative necrosis of the target tissue	Cavitation bubbles form in the target area, and the bubbles expand and collapse violently, mechanically tearing cells into fragments.
Pulse duration	Continuous or long pulses	Short pulses (1–2 cycles)
Frequency	0.2–2 MHz	250 kHz–5 MHz
Duty Cycle	High (often continuous)	<2%

**Figure 1:**
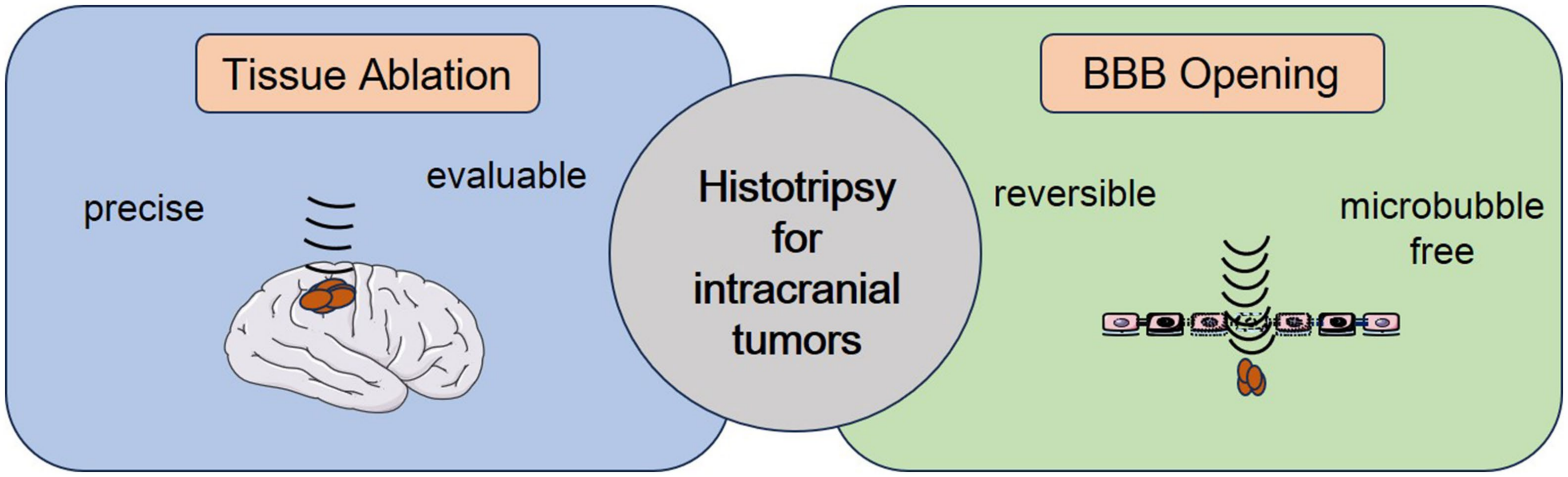
Schematic illustration of histotripsy application for intracranial tumor therapy.

Histotripsy is a noninvasive, nonthermal, nonionizing ultrasonic tissue-ablation technology. It utilizes megapascal-scale, high-amplitude ultrasound pulses to trigger inertial cavitation in the target area (10). During the collapse of cavitation bubbles, intense mechanical stresses are released. This leads to cell membrane rupture and subcellular structure disintegration (11). Current preclinical studies have demonstrated that histotripsy can precisely ablate tissue and controllably open the blood–brain barrier (BBB) (Figure 1). These diverse therapeutic effects give histotripsy unique potential in intracranial tumor treatment.

## 2 Tissue ablation

### 2.1 Mechanical ablation of tumor and normal brain tissue

Histotripsy encompasses multiple types—including intrinsic threshold, shock-scattering, and boiling histotripsy. Intrinsic threshold histotripsy relies on a single, focused ultrasound pulse of sufficient pressure to directly exceed the intrinsic tensile strength of the tissue. Shock-scattering histotripsy, in contrast, employs a lower-amplitude pulse sequence that leverages the interaction between shockwaves and pre-existing or incident cavitation nuclei, enabling a “cascade” or “relay-like” growth of the cavitation cloud. Boiling histotripsy represents a distinct hybrid approach: it first utilizes the shockwave heating within the focus to generate a millimetric vapor bubble, which then serves as the nucleus for subsequent mechanical cavitation and tissue disruption (12). Despite operating through distinct physical mechanisms, various types of histotripsy collectively achieve precise and selective tumor ablation while preserving the structural integrity of surrounding healthy tissue (9). The ablation process selectively spares structures with higher mechanical stiffness, such as blood vessels and bile ducts, due to their significantly elevated Young’s modulus compared to adjacent soft tissue. Moreover, the comparatively lower stiffness of malignant cells renders them preferentially susceptible to non-thermal, cavitation-induced mechanical disruption. Consequently, histotripsy generates lesions with exceptionally sharp demarcation between ablated and intact tissue, a critical feature for achieving precise surgical margins while sparing vital structures (13, 14). Ex vivo experiments using human skull models have confirmed the efficacy of transcranial histotripsy in generating cavitation clouds and effecting precise tissue ablation (15). In canine meningioma models, despite some degree of acoustic pressure attenuation, MRI-guided histotripsy produced well-defined ablation boundaries. Histopathological analysis revealed uniformly liquefactive necrosis within the target zone without involvement of adjacent neurovascular structures (16). In all porcine models, Lu et al. consistently achieved successful and precise tissue ablation via histotripsy. Magnetic resonance imaging confirmed that the ablation zone was strictly confined to the target volume, with no evidence of significant perilesional edema or extra-target hemorrhage. Histological analysis further demonstrated homogeneous homogenization of the ablated tissue and a sharp demarcation between the disrupted region and the surrounding unaffected parenchyma (17, 18).

In addition, precise pretreatment targeting ensures the accurate delivery of histotripsy energy to the intended focal point, which is critical for both treatment efficacy and safety. Gupta et al. systematically addressed the critical challenge of pretreatment targeting for transcranial histotripsy. Their experimental protocol, utilizing ex vivo bovine brains, involved ablation at 35 MPa, 75 MPa, and through the skull at 36 MPa (19). These researchers implemented MR-ARFI and MR-thermometry for preoperative focal spot localization and rigorously validated the targeting accuracy by correlating the predicted locations with the resultant histotripsy lesions. This study provides the foundational evidence that MR-ARFI and MR-thermometry are viable and accurate methods for ensuring precise energy delivery in transcranial histotripsy, thereby paving the way for its reliable clinical translation (19). Although current preclinical evidence remains limited by small sample sizes and short observation periods, it has preliminarily established a foundation for both feasibility and safety. Future studies should focus on expanding cohort sizes and validating long-term safety to address these limitations and facilitate translation from bench to bedside.

### 2.2 Evaluation of ablation effect

Histotripsy is a non-thermal ablation technology that mechanically disrupts brain tumors via acoustic cavitation. Monitoring of the ablation process is an essential prerequisite for ensuring its treatment efficacy. To address this need, several innovative approaches have recently been established. Gupta et al. pioneered a modified Gradient Echo (GRE) sequence, termed MR-Cavitation Dynamics Encoded (MR-CaDE) imaging, which integrates a bipolar motion-encoding gradient to detect histotripsy-induced cavitation. Implemented on a clinical 3 T scanner in ex vivo bovine brains, MR-CaDE achieved a temporal resolution of 0.5 s, enabling near real-time observation of characteristic signal dynamics—a progressive decrease in magnitude coupled with a phase increase—that correlated directly with sonication number. This work definitively establishes MR-CaDE as a potent non-invasive tool for quantifying cavitation activity (20). Complementing this, Choi et al. provided the critical radiological-histopathological correlation necessary for interpreting such imaging data. Through longitudinal MRI and terminal histology in mouse models, they systematically defined the temporal evolution of imaging features that mirror specific treatment-induced tissue changes, creating a validated framework for non-invasive assessment of histotripsy effects *in vivo*. Histotripsy induces limited focal hemorrhage, which undergoes complete resolution within 7 days as observed in hematoxylin and eosin (H&E)-stained sections. By day 14, the ablation zone is delineated solely by a peripheral rim of hemosiderin-laden macrophages (21). Moreover, Sukovich et al. (22) demonstrated a paradigm-shifting, non-MRI-based methodology for real-time cavitation monitoring. Their technique leverages the intrinsic acoustic cavitation emission (ACE) signals, detected in receive-mode by the very same 256-element therapeutic transducer array, to localize and map the cavitation cloud through an intact human skullcap. This represents a significant engineering advance, offering a practical and accessible alternative to MRI for real-time treatment monitoring (22). While the ex vivo nature of some findings necessitates further *in vivo* validation, the convergence of these MR-based and acoustic monitoring technologies is paving the way for reliable, image-guided application of transcranial histotripsy, marking a pivotal step toward its successful clinical translation for intracranial tumor therapy.

## 3 Histotripsy-mediated opening of the BBB

The blood–brain barrier (BBB) presents a dual-pathway obstacle to neuro-oncological therapy, severely compromising chemotherapeutic efficacy through both physical and biochemical exclusion mechanisms. Its tightly sealed endothelial junctions impede paracellular diffusion, while ATP-binding cassette (ABC) transporters—including P-glycoprotein and MRP1—actively efflux drugs, resulting in exceptionally low brain bioavailability (23, 24). Histotripsy has emerged as a promising strategy to overcome these delivery barriers. One important effect of histotripsy in the brain is the blood–brain barrier (BBB) opening (BBBO) at the ablation site, but there is a knowledge gap concerning the extent of histotripsy-induced BBBO. While permanent BBB disruption occurs within the fully ablated core, the peri-focal region experiences a transient, reversible opening—a phenomenon whose spatiotemporal dynamics had remained inadequately characterized (25). Duclos et al. (25) systematically quantified this process using longitudinal MRI and histology, demonstrating that the volume of BBB opening (BBBO), visualized by T1-Gd hyperintensity, expands during the first post-treatment week and gradually resolves over the subsequent 3 weeks. Histological analysis revealed an immediate and complete loss of tight junction proteins (e.g., claudin-5, ZO-1) and vasculature in the ablation core, followed by partial recovery in the periphery by week 1 and near-complete restoration of the junctional complex by week 4 (25). These findings, corroborated by contrast-enhanced MRI and immunohistochemical evidence of transient TJP downregulation with subsequent recovery, establish histotripsy as a potent modality for achieving spatiotemporally controlled BBB opening. This capacity for targeted and reversible barrier modulation creates significant potential for enhancing drug delivery to brain tumors and other central nervous system pathologies.

Unlike conventional focused ultrasound combined with exogenous microbubbles (FUS + MB), histotripsy operates through endogenous cavitation nuclei within the tissue, eliminating the need for exogenous microbubbles (25, 26) (Table 2). This represents a novel strategy for precise and microbubble-independent BBB modulation, though parameter optimization remains essential for clinical translation. Furthermore, combining histotripsy-mediated BBB opening with chemotherapy or immunotherapy may bring a transformative approach for the treatment of intracranial tumors.

**Table 2:** Comparison between FUS + MB and histotripsy in opening the blood–brain barrier [data is from the references (50–52)].

Comparison dimensions	Microbubble-mediated FUS for BBBO	Histotripsy for BBBO
Core mechanism	Stable cavitation	Inertial cavitation
Working principle	Microbubble oscillation generates shear stress, reversibly disrupting tight junctions.	Rapid expansion/collapse of cavitation bubbles generates high strain/stress, mechanically fractionating tissue.
Ultrasound parameters	Low peak pressure (0.3–1.5 MPa), long pulses (10–100 ms)	High peak pressure (>20 MPa), short pulses (microsecond length)
Microbubble requirement	Required	Not required
Effect on BBB	Temporary, selective opening	Permanent disruption (at focus center), reversible opening (at focus periphery)
Technical Status	In clinical trials	Primarily in the preclinical research stage

## 4 General immune effects of histotripsy

### 4.1 Local immune modulation

Histotripsy, a non-thermal ablation modality based on mechanical cavitation, induces liquefactive necrosis while eliciting profound and distinct immunomodulatory effects (27). Gerhardson et al. (28) demonstrated in a murine GL261 intracranial glioma model that partial histotripsy ablation of the brain tumor induces a pronounced immunomodulatory response within the tumor microenvironment, characterized by a significant upregulation of interferon-gamma and a concomitant reduction in myeloid-derived suppressor cells in the brain tissue. Pahk et al. (29) found that histotripsy can trigger immunogenic cell death primarily through a TNF-α-mediated necroptotic pathway. This process promoted substantial release of damage-associated molecular patterns (DAMPs)—including calreticulin, HSP-70, and HMGB-1—along with pro-inflammatory cytokines (IFN-γ, IL-1α, IL-1β, IL-18) and the chemokine IL-8, collectively establishing an immunogenic microenvironment conducive to M1 macrophage polarization. Notably, the intensity of this immune signaling cascade exhibited direct correlation with the degree of histotripsy-induced tissue damage.

Mechanistically, the non-thermal nature of histotripsy preserves the immunogenicity of tumor antigens by avoiding protein denaturation—a distinct advantage over thermal ablation modalities (30). This preservation enables cellular debris to function as potent endogenous vaccines, thereby amplifying subsequent immune recognition and response. The enhanced TNF-α signaling not only reinforces the immunogenic cell death cycle but also significantly upregulates key immunomodulators, including HSP-70, IFN-γ, and IL-18, establishing a positive feedback loop that sustains antitumor immunity (31).

### 4.2 Abscopal effects

The abscopal effect describes the phenomenon in which histotripsy is capable of eliciting anti-tumor immune responses at sites distant from the primary treatment target. In tumor-bearing models, treatment not only enhances local immune infiltration but also activates systemic immunity, evidenced by increased CD8^+^ T cell presence in untreated regions (32). Similarly, research by Worlikar et al. (33) indicates that even partial histotripsy ablation can ultimately lead to regression of non-targeted tumor regions. This effect is likely attributable to enhanced immune infiltration into distant tumor sites following treatment (33). Hu et al. (34) demonstrated that histotripsy significantly enhanced dendritic cell activation in tumor-draining lymph nodes, elevating CD11c^+^ cell and mature DC populations by 1.3-fold and 2-fold, respectively, compared to thermal ultrasound ablation. Pepple et al. (35) further suggest that histotripsy triggers immunogenic necroptosis locally, thereby priming a systemic adaptive immune response and potentially inducing abscopal ferroptosis in distant tumors. These results establish histotripsy as a mechanistically distinct immunotherapeutic strategy, capable of eliciting robust systemic immune activation beyond the limitations of thermally based modalities, thereby positioning it as a promising platform for combination oncology regimens.

While preclinical data confirm that histotripsy inhibits the growth of non-targeted tumors and prolongs survival, key mechanistic aspects require further elucidation. These include the dynamics of antigen cross-presentation, DAMP-mediated innate immune activation, and reprogramming of immunosuppressive microenvironments. Current evidence, primarily from small-scale animal studies, leaves the consistency and reliability of the abscopal effect incompletely resolved. Future work should focus on validating its reproducibility across models and establishing biomarker-guided predictive frameworks to stratify treatment response, thereby advancing this promising modality toward clinical application.

### 4.3 Synergy with ICIs

Furthermore, emerging approaches that integrate histotripsy with immune checkpoint inhibitors (ICIs) demonstrate particular promise, leveraging mechanical disruption to potentiate immunotherapeutic outcomes (36). The limited efficacy of immunotherapy in intracranial tumors, characterized by suboptimal response rates to ICIs, underscores the need for innovative combination strategies. Imran et al. demonstrate that the “magic bubble” phenomenon—cavitation bubbles generated during histotripsy—not only mechanically fractionates tumors but also initiates potent innate immune activation. This dual action promotes dendritic cell maturation and systemic CD8^+^ T cell priming, effectively initiating adaptive immunity. Notably, this immunologically activated state unlocks a unique immunotherapeutic axis, demonstrating marked synergy with checkpoint inhibitors and offering a compelling strategy to counteract immunosuppressive tumors through histotripsy-immunotherapy combinations (37). These studies position histotripsy as a potent “sensitizer” capable of converting immunologically inert tumors into targets that are vulnerable to ICIs. While direct preclinical evidence of the histotripsy-ICI combination for intracranial tumors is currently lacking, the established mechanistic data provide a compelling rationale and clear direction for future investigation. While challenges persist—including the durability of response, heterogeneity of the tumor microenvironment, and optimization of treatment schedules—the precision of histotripsy energy delivery enables localized immune remodeling even in deep-seated brain regions. This unique capability to mechanically reconfigure the immunosuppressive landscape presents a novel therapeutic paradigm for overcoming immunotherapy resistance in neuro-oncology, potentially expanding the applicability of ICIs to previously refractory intracranial malignancies.

**Figure 2:**
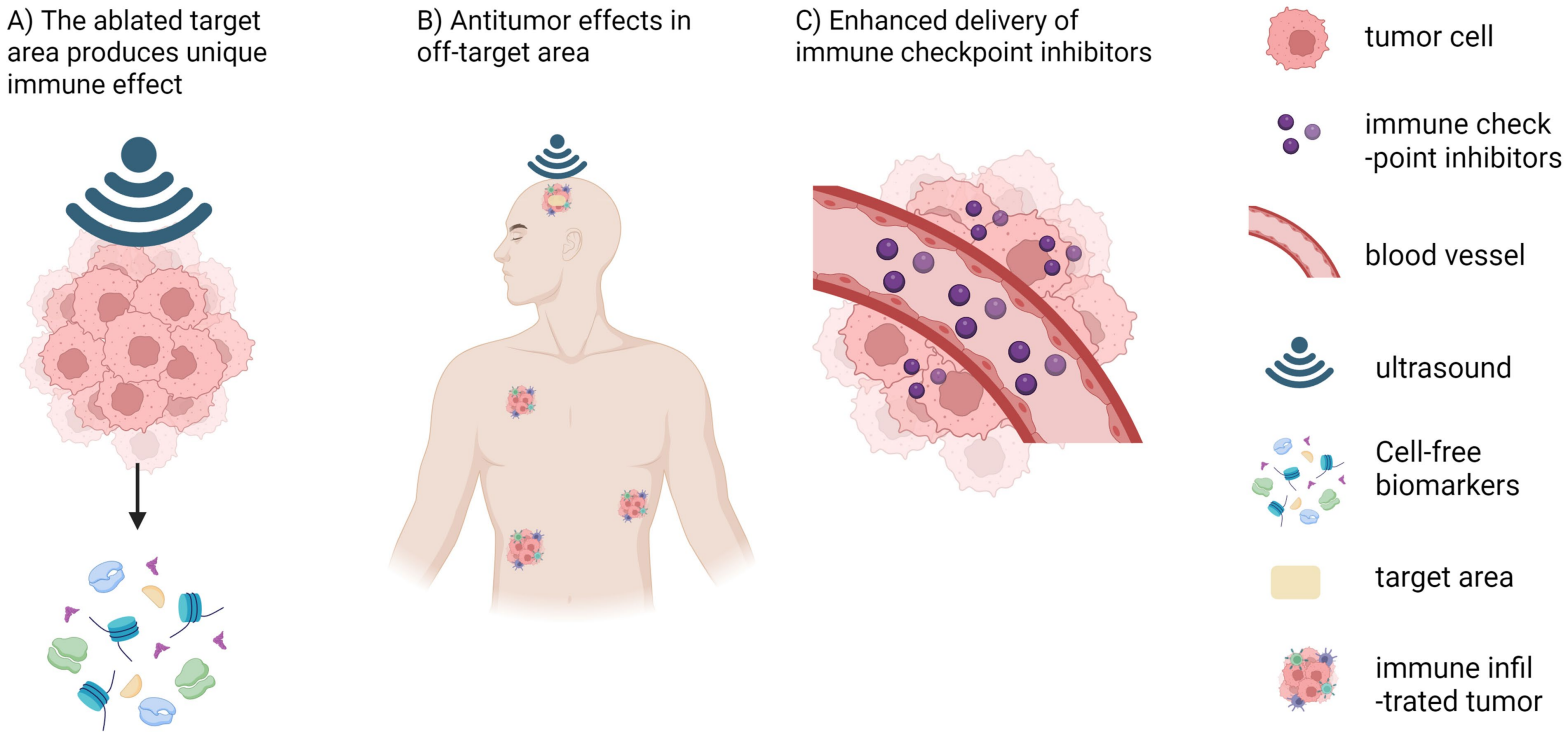
Proposed mechanisms underlying the general immune effects of histotripsy, including **(A)** The ablated target area produces unique immune effect, **(B)** Antitumor effects in off-target area, **(C)** Enhanced delivery of immune checkpoint inhibitors. Created in BioRender. Chen, H. (2025) https://BioRender.com/t3lfeg6.

In summary, histotripsy’s multimodal immune effects include three aspects: (1) liquefactive necrosis induced by histotripsy can produce unique immunological effects; (2) histotripsy can induce immune responses in non-target areas (abscopal effect); and (3) the combination of histotripsy and immune checkpoint inhibitors can synergistically activate antitumor immune responses (Figure 2). To sum up, a growing body of preclinical evidence substantiates that histotripsy can elicit pro-inflammatory immunomodulation within the tumor microenvironment—an effect with considerable therapeutic promise. However, direct experimental evidence demonstrating its capacity to initiate or augment an antitumor immune response specifically against intracranial tumors remains absent. Addressing this critical knowledge gap through dedicated future research is not only warranted but essential to evaluate the full translational potential of histotripsy-immunotherapy combinations for brain tumors.

## 5 Aberration correction of transcranial histotripsy

Aberration correction remains a pivotal technical challenge in the transcranial application of histotripsy for intracranial tumor therapy. Current research focuses on several advanced compensation strategies: (1) Two-Step Aberration Correction Strategy: A hybrid aberration correction method was developed, combining an initial CT-based analytical prediction with subsequent refinement using shockwaves derived from acoustic cavitation emissions (ACE). This two-step approach demonstrated superior focal precision compared to either CT-based or ACE-based correction alone and can be executed in real time, offering a practical solution for transcranial histotripsy therapy (38). (2) Implantable Acoustic Windows: an Acoustically Permeable Polyolefin-Based Cranioplasty Device has been proposed. It can function as an acoustic window, reducing pressure attenuation of histotripsy caused by the skull and providing a stable sound channel for non-invasive treatment (39). (3) Advanced Array Design: A specialized ultrasound array was designed to generate high-amplitude shock fronts at the focus through precise modeling of wave propagation. Simulations integrated three numerical algorithms—Rayleigh integral for source modeling, a linear pseudo-spectral time-domain Kelvin–Voigt model for viscoelastic skull effects, and a nonlinear Westervelt model—to comprehensively account for skull-induced aberrations, reflections, absorption, nonlinearity, and shear waves (40). (4) Time-Reversal Algorithm: Based on pre-operative CT data, this method calculates skull-dependent phase distortions from thickness and density variations, enabling phase compensation via acoustic wave reconstruction (41). (5) Transmission Matrix Approach: The acoustic transmission matrix between each transducer element and the focus is empirically measured. Matrix inversion or adaptive algorithms then derive driving signals to pre-compensate for skull-induced distortions (42).

## 6 Current challenges and future prospects

To date, histotripsy for intracranial tumors remains in the preclinical stage. Current trials mainly focus on safety and feasibility, lacking sufficient samples and proper controls to measure efficacy. However, preclinical experiments have confirmed histotripsy’s effectiveness for intracranial tumors (Table 3). Also, clinical evidence of histotripsy in other diseases has achieved significant breakthroughs. For instance, Mendiratta-Lala et al. (43) conducted a prospective, multicenter clinical trial demonstrating the safe and efficacious ablation of targeted liver tissue using histotripsy, providing compelling evidence to support its clinical adoption for the treatment of liver tumors.

**Table 3:** Overview of partial preclinical studies on histotripsy for intracranial tumors with relatively high impact factor in recent years.

Tumor type	Experimental animal	Transducer frequency	Pulse Repetition Frequency	Peak negative pressure	Pulse length	Model type	Main effects	References
Glioblastoma	Mouse	1 MHz	5 Hz	36 MPa	1.5 cycles	*In vivo*	Reversible opening of the blood–brain barrier.	(25)
		1.5 MHz	10 Hz	87 MPa	3 cycles	*In vivo*	Mechanically disrupt the target tissue.	(53)
	Pig	500 kHz	0.5 Hz	48 MPa	NA	*Ex vivo*	Mechanically disrupt the target tissue.	(54)
Meningioma	Canine	1 MHz	100 Hz	40.5 MPa	1 cycle	*In vivo*	Significantly increased HMGB1 expression.	(16)

The substantial capital investment required for histotripsy systems, largely attributable to complex multi-element transducer arrays, constitutes a significant barrier to their near-term widespread clinical adoption. Future cost-reduction strategies may involve employing generative adversarial networks (GANs) to simulate complex tissue acoustic environments, thereby optimizing array design and reducing the required number of elements without compromising performance (19). At present, transcranial histotripsy uses big, costly, and inflexible transducers. Future device configuration and form-factor optimization is needed. Some researchers have proposed combining endoscopy with histotripsy for natural-orifice access, enabling dynamic focus-depth adjustment of 3–8 mm. This would better match minimally invasive neurosurgical procedures and reduce healthy-tissue damage (44). A miniaturized histotripsy transducer has been reported, which achieves a critical gain in peak negative pressure through the synergistic combination of an acoustic lens and dual-frequency stacked elements. This integrated design enables potent cavitation generation from a compact form factor, unlocking new potential for minimally invasive ablation procedures (45).

A critical barrier to the clinical translation of transcranial histotripsy is the absence of a robust, real-time guidance modality. To address this, Choi et al. (46) recently developed the first neuronavigation-guided transcranial histotripsy system and its associated clinical workflow. The platform integrates a 700 kHz, 360-element therapeutic array capable of both transmission and reception, a clinical-grade neuronavigation system, and dedicated software for patient-to-array co-registration, therapy planning, and dynamic targeting. This integrated technological framework establishes a pathway toward precise transcranial ablation without the dependency on intraoperative magnetic resonance imaging for real-time guidance (46). Furthermore, histotripsy has demonstrated a promising capacity to enhance local chemotherapeutic delivery in preclinical models of extracranial tumors. Key evidence includes a study reporting a markedly elevated intratumoral concentration of doxorubicin within the histotripsy-treated region compared to untreated controls (47). However, whether this chemo-potentiating effect translates to intracranial tumors remains an open question. Investigating the impact of histotripsy on chemotherapy for brain tumors represents a critical and logical next step, with the potential to establish a novel combined modality for these challenging cancers. While techniques such as Nakagami imaging have demonstrated potential for preliminary assessment of blood–brain barrier (BBB) disruption, the establishment of a validated, precise algorithm for the real-time quantification of drug delivery following BBB opening remains an unmet need, currently limiting the precision and efficacy of combined pharmaco-histotripsy strategies (48). Current researches have largely focused on the ablation effects of histotripsy. Iwanicki et al. (49) reported that beyond its direct mechanical ablation, histotripsy can also induce multifaceted antitumor effects, drive tumor cell death via the intrinsic apoptotic pathway, concurrently remodels tumor vasculature, and alleviates intratumoral hypoxia. Deciphering the relative contributions and interplay of these mechanisms represents a critical frontier for future research, with significant implications for optimizing combination therapies (47). In conclusion, despite existing challenges in transcranial focusing and economic accessibility, histotripsy holds considerable promise for the treatment of brain tumors, particularly through continued innovation in aberration correction, cost reduction, and integrative therapeutic strategies.

## 7 Conclusion

In summary, histotripsy represents a groundbreaking non-invasive platform that achieves precise tumor ablation through mechanically induced cavitation. Histotripsy enables precise, evaluable ablation of brain tissue. Furthermore, it facilitates a novel, non-invasive, and microbubble-free strategy for transient and reversible blood–brain barrier opening, thereby establishing a possible therapeutic window for targeted drug delivery. In the field of precision medicine, histotripsy, with its non-thermal and immune-modulating advantages, is creating a new clinical paradigm for physical-energy-based treatments. While histotripsy for intracranial tumors is still in preclinical stages, phase II trial data from other types of tumors (such as hepatocellular carcinoma) prove its therapeutic potential. Histotripsy thereby establishes itself as a truly disruptive platform technology, poised to redefine the therapeutic landscape for a spectrum of oncological diseases.
